# Intranasal Ectopic Tooth in an Adolescent With Unilateral Cleft Lip and Palate: A Case Report

**DOI:** 10.1002/ccr3.73836

**Published:** 2026-10-30

**Authors:** Mikyas Befekadu Nigusse, Bewuketu Kefyalew Zewudie, Ermias Algawork Kibru, Nuhamin Daniel Kiflemariam, Bemnet Abi Zeleke

**Affiliations:** ^1^ Department of Radiology MCM Comprehensive Specialized Hospital Addis Ababa Ethiopia; ^2^ Myungsung Medical College Addis Ababa Ethiopia; ^3^ Department of Radiology Piedmont Athens Regional Hospital Athens Georgia USA

**Keywords:** adolescent, cleft lip and palate, computed tomography, facial pain, intranasal tooth, supernumerary tooth

## Abstract

A 14‐year‐old male with repaired cleft lip and palate presented with unilateral foul‐smelling nasal discharge, obstruction, and facial pain. CT identified an intranasal ectopic tooth with chronic sinusitis. Surgical removal using a Caldwell Luc approach resulted in symptom resolution. Early diagnosis and imaging are crucial for effective management and preventing complications.

## Key Clinical Message

This case highlights an intranasal ectopic tooth, a rare anomaly occasionally associated with cleft lip and palate. In resource‐limited settings, delayed access to computed tomography (CT) may delay diagnosis. CT is essential for diagnosis and surgical planning, while early removal prevents complications and improves outcomes.

## 1 Introduction

Intranasal teeth are rare developmental anomalies characterized by ectopic eruption of a tooth within the nasal cavity. Due to their uncommon nature, only a limited number of cases have been reported in the literature [1]. Clinical presentation varies, with some patients remaining asymptomatic while others develop nasal obstruction, epistaxis, facial pain, or foul‐smelling discharge [2]. The diagnosis is based on clinical assessment and imaging, with computed tomography (CT) being the preferred modality for confirming the diagnosis, defining tooth location, detecting associated craniofacial abnormalities, assessing sinonasal complications, and guiding surgical planning [3]. Surgical removal is indicated in symptomatic cases or when complications develop to relieve symptoms and prevent further morbidity [4]. Awareness of this condition, its clinical features, and radiologic characteristics is essential for accurate diagnosis and appropriate management in craniofacial patients.

## 2 Case History and Examination

A 14‐year‐old male who presented with a history of left‐sided cleft lip and palate defect, for which he had undergone primary reparative surgery. According to the patient and his family, cleft lip repair was performed at 6 months of age, followed by palatal repair at 4 years of age. Due to limited access to specialized cleft care services in the local setting, the repairs were performed later than the commonly recommended age range. The surgeries were reportedly performed through a foreign aid program in local hospital; however, details regarding the specific surgical techniques used and any additional procedures performed were unavailable. Following repair, there was significant improvement in feeding difficulties and overall function.

Despite the initial improvement, the patient continued to experience recurrent episodes of unilateral foul‐smelling nasal discharge, persistent nasal obstruction, and intermittent facial pain. These symptoms temporarily improved with repeated courses of oral antibiotics but recurred over several years. Given his history of repaired cleft lip and palate (CLP), his chronic sinonasal symptoms were initially attributed to the altered sinonasal anatomy associated with CLP.

Due to the persistent and recurrent nature of her symptoms, He was referred to the otorhinolaryngology service at a governmental hospital in Addis Ababa for further evaluation. A detailed assessment with CT of the nasal cavity and paranasal sinuses was requested to evaluate possible structural causes of her chronic symptoms and to guide surgical management.

On physical examination, intraoral examination revealed permanent dentition with mandibular anterior crowding and a missing left maxillary incisor. An endodontically treated maxillary first permanent molar was noted. The patient had a history of left‐sided repaired cleft lip and palate with a residual left alveolar cleft (Figure 1). No clinically evident oronasal fistula was identified. Anterior rhinoscopy and nasal endoscopy were not performed.

**FIGURE 1:**
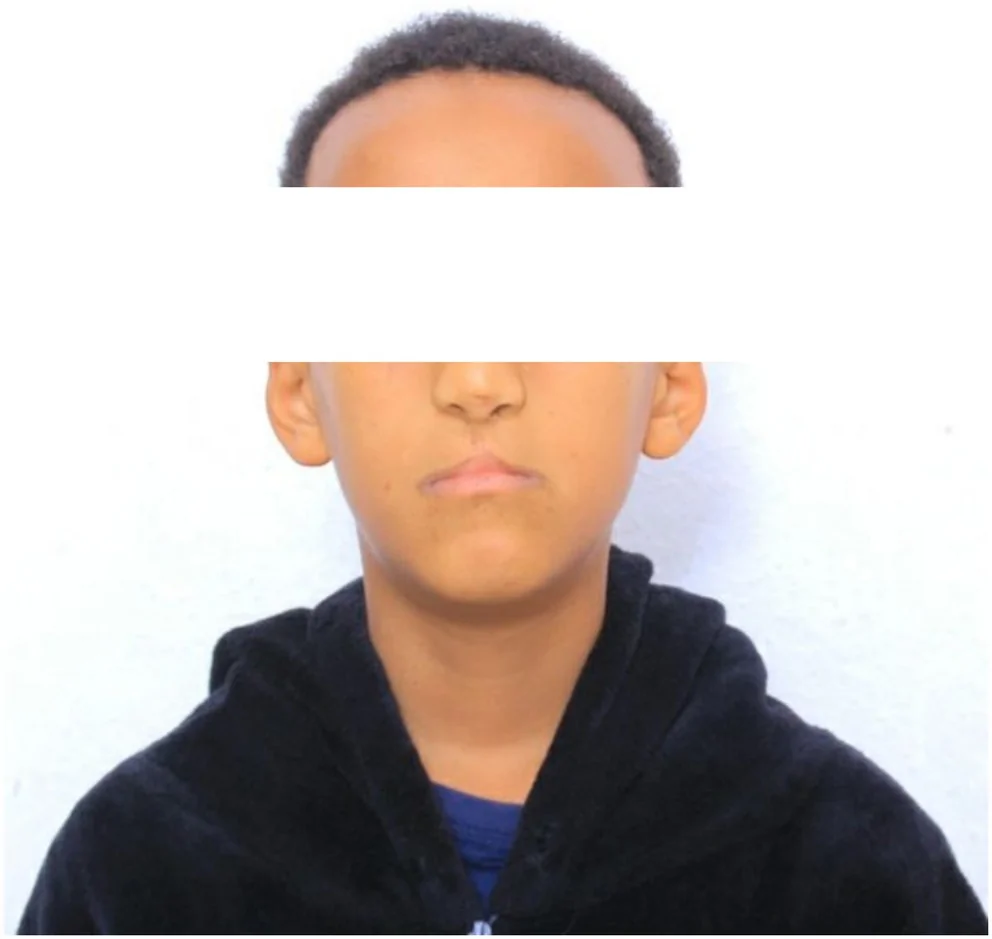
Preoperative image showing a patient who had repaired left cleft lip and palate.

## 3 Methods

### 3.1 Investigation

Routine laboratory investigations were within normal limits. The CT scan demonstrates a left‐sided unilateral osseous defect involving the alveolar process of the maxilla and the hard palate, filled with postsurgical soft tissue, consistent with a repaired CLP (Figures 2 and 3). A well‐defined hyperdense structure is seen within the left nasal cavity, attached to the mucosal surface of the lateral wall of the left nostril. The lesion has the attenuation characteristics and morphology of a tooth crown, with attenuation values ranging from 2140 to 2189 HU (Figures 4 and 5). Three‐dimensional volume‐rendered images (Figures 6 and 7) more clearly demonstrate that this intranasal structure is an ectopic tooth. It has a single root oriented cranially and a crown directed inferiorly, with an orientation similar to that of the adjacent maxillary teeth. Additionally, mucosal thickening, sinus opacification, and fluid‐attenuation collections are present in the left maxillary and ethmoid sinuses, consistent with chronic sinusitis, likely secondary to the underlying cleft lip and palate.

**FIGURE 2:**
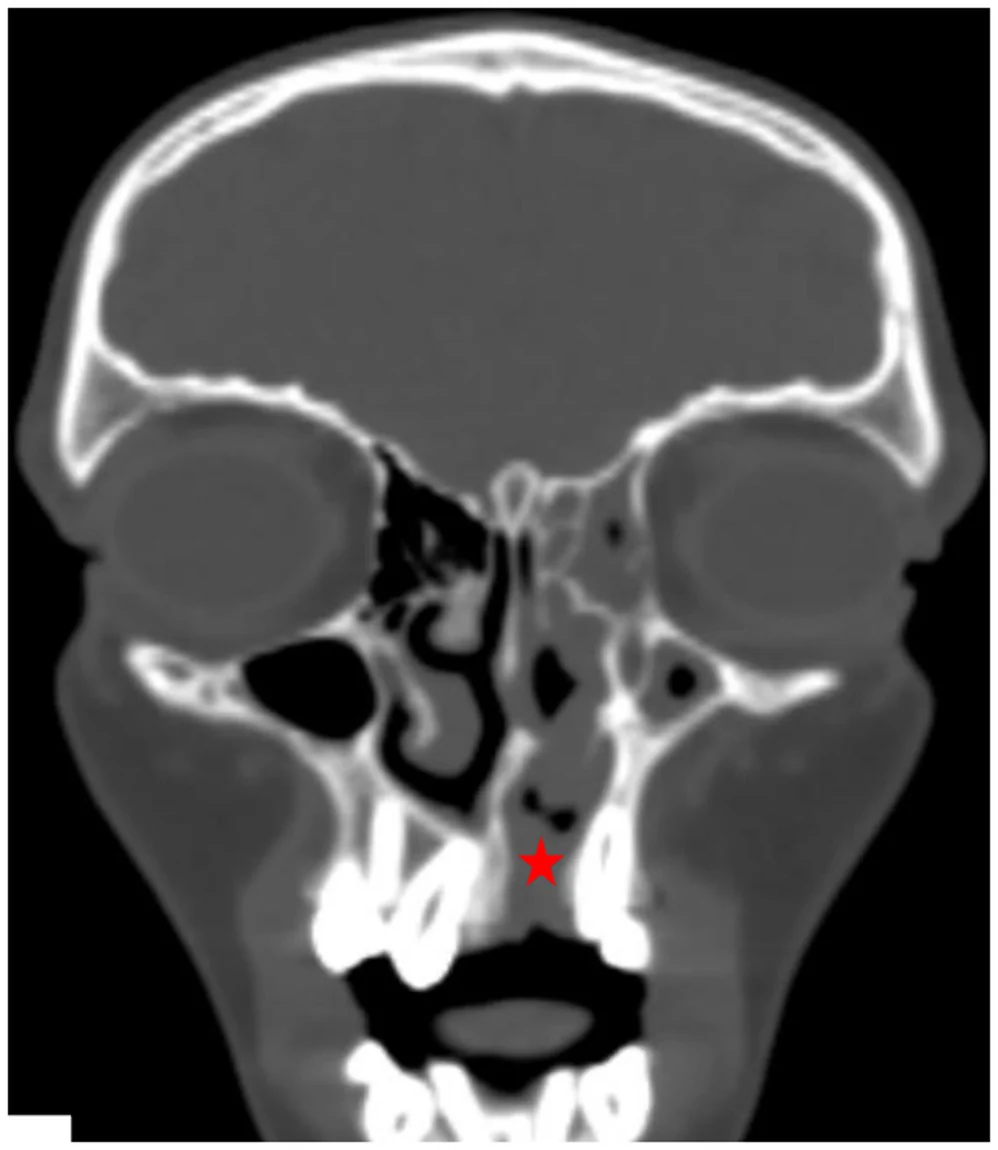
Coronal bone‐window CT image demonstrating a left‐sided unilateral bone defect of the alveolar process of the maxillary bone (asterisk).

**FIGURE 3:**
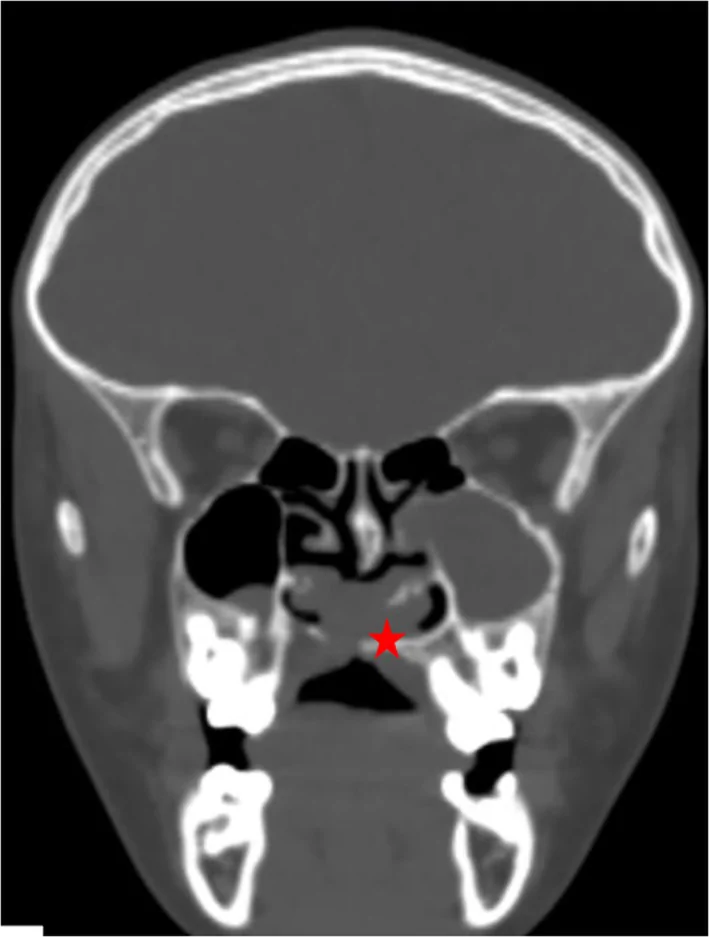
Coronal bone‐window CT image at a different level demonstrating extension of the left alveolar defect into the hard palate (asterisk); consistent with a cleft lip and palate deformity. The soft tissue density material filling the bone defect is post‐surgical repair. The associated left maxillary and ethmoid sinuses' mucosal thickening and fluid attenuating collection associated with chronic sinusitis (a common complication in patients with CLP).

**FIGURE 4:**
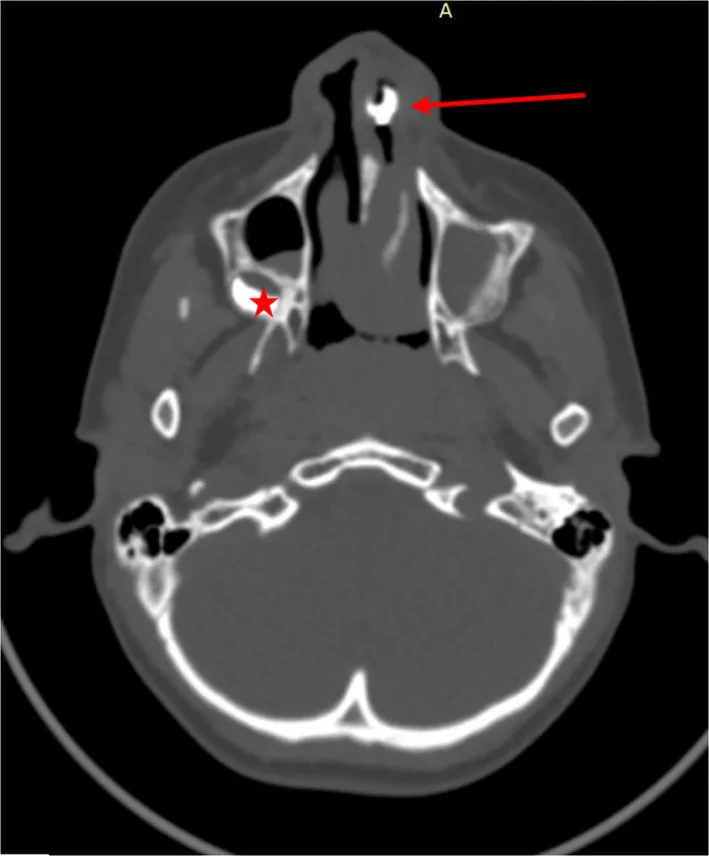
Axial bone‐window CT image showing a well‐defined hyperdense material (arrow) within the left nasal cavity attached to the lateral nasal wall with the same density and attenuation value of the maxillary tooth (asterisk).

**FIGURE 5:**
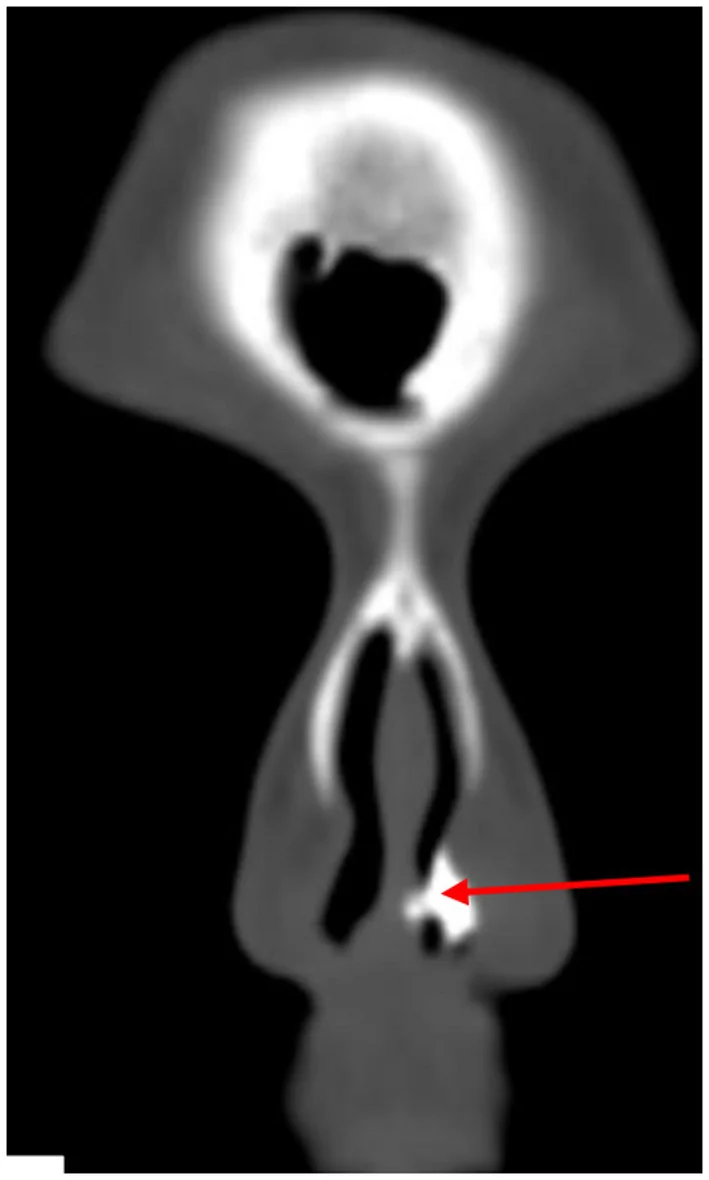
Bone window coronal CT image of the same patient at the level of the nares showing the orientation of the tooth as the root points upward and is attached to the lateral wall while the crown is directed downward, similar to the maxillary teeth.

**FIGURE 6:**
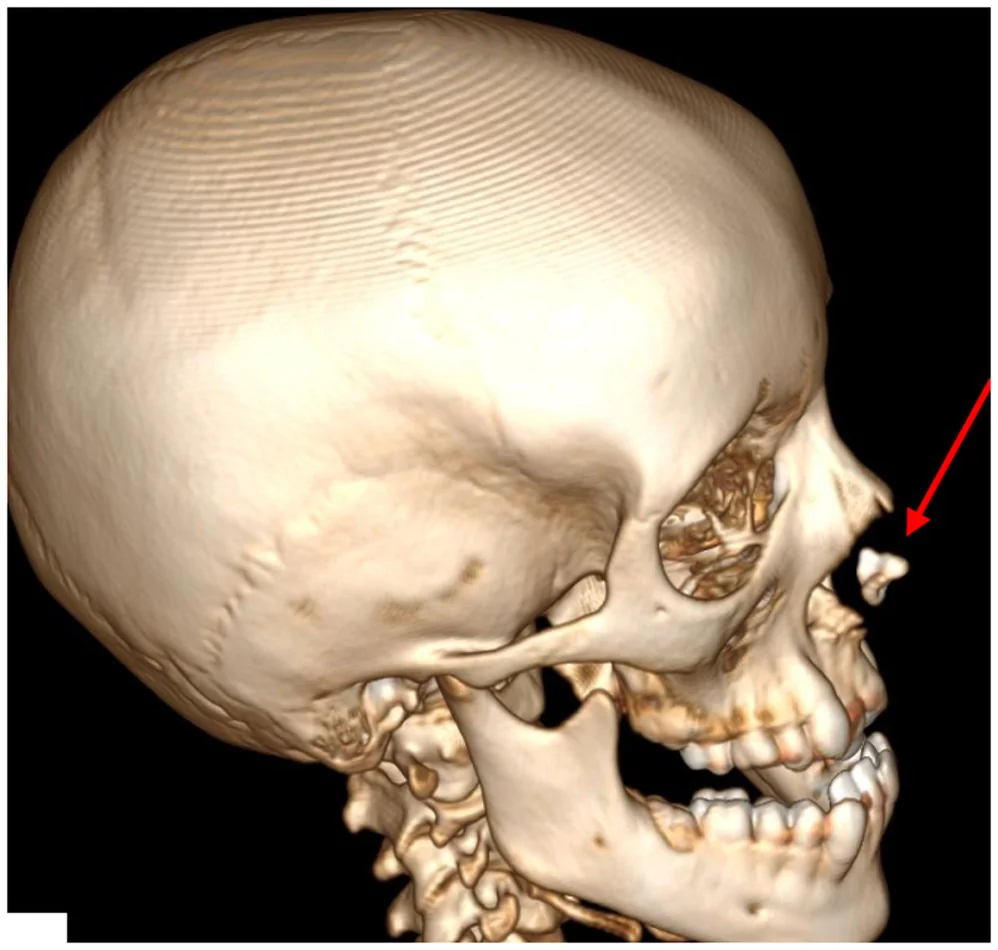
Volume rendering CT image of the head better demonstrating the orientation and tooth nature of the intranasal structure.

**FIGURE 7:**
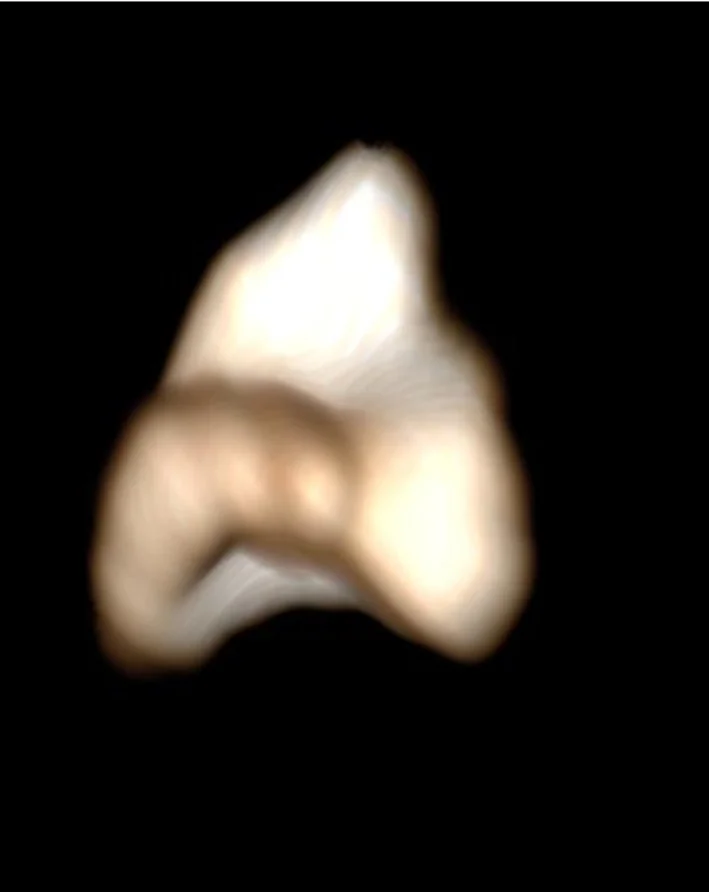
Magnified image of the tooth removed from the vomer. The image clearly demonstrates both the crown and the root.

### 3.2 Differential Diagnosis

The differential diagnosis of a radiopaque intranasal lesion included foreign body, rhinolith, bone sequestrum, neoplasm, and exostosis. These were excluded based on the characteristic dental morphology, including a recognizable crown and root configuration, orientation similar to adjacent teeth, and high attenuation values consistent with dental hard tissue. Unlike foreign bodies or rhinoliths, the lesion demonstrated an organized tooth‐like structure rather than irregular calcification. Bone sequestrum and exostosis were unlikely due to the absence of continuity with surrounding bone, while neoplasm was excluded by the lack of associated soft tissue mass or destructive features. The diagnosis was subsequently confirmed following surgical extraction of the specimen.

### 3.3 Treatment

The patient underwent surgical removal of the ectopic tooth through a Caldwell–Luc approach. This approach was selected instead of a purely endoscopic endonasal approach because of the anterior/lateral location of the tooth and the altered anatomy associated with repaired cleft lip and palate. The procedure was performed by an otorhinolaryngology (ENT) surgeon through a combined intraoral and intranasal approach. Following a gingivobuccal incision in the canine fossa, a bony window was created to access the maxillary antrum. The tooth was carefully mobilized from the lateral nasal wall and extracted without injury to adjacent structures. The maxillary sinus was irrigated, inflamed mucosa was debrided as required, hemostasis was achieved, and the incision was closed in layers. No intraoperative complications occurred.

### 3.4 Outcome and Follow‐Up

Postoperatively, the patient received appropriate analgesia, antibiotics, and nasal saline irrigation. Considering the altered anatomy related to previous cleft repair, the anterior location of the ectopic tooth, and the available surgical expertise, the Caldwell–Luc approach was selected as a reliable method for controlled removal. Following surgery, the patient demonstrated marked clinical improvement. Nasal obstruction resolved, the foul‐smelling nasal discharge subsided, and facial discomfort improved (Figure 8). He was discharged in good clinical condition. This case emphasizes the importance of careful radiologic evaluation in patients with repaired cleft lip and palate who present with persistent sinonasal symptoms and highlights the role of surgical removal of ectopic teeth as a definitive treatment for symptom resolution.

**FIGURE 8:**
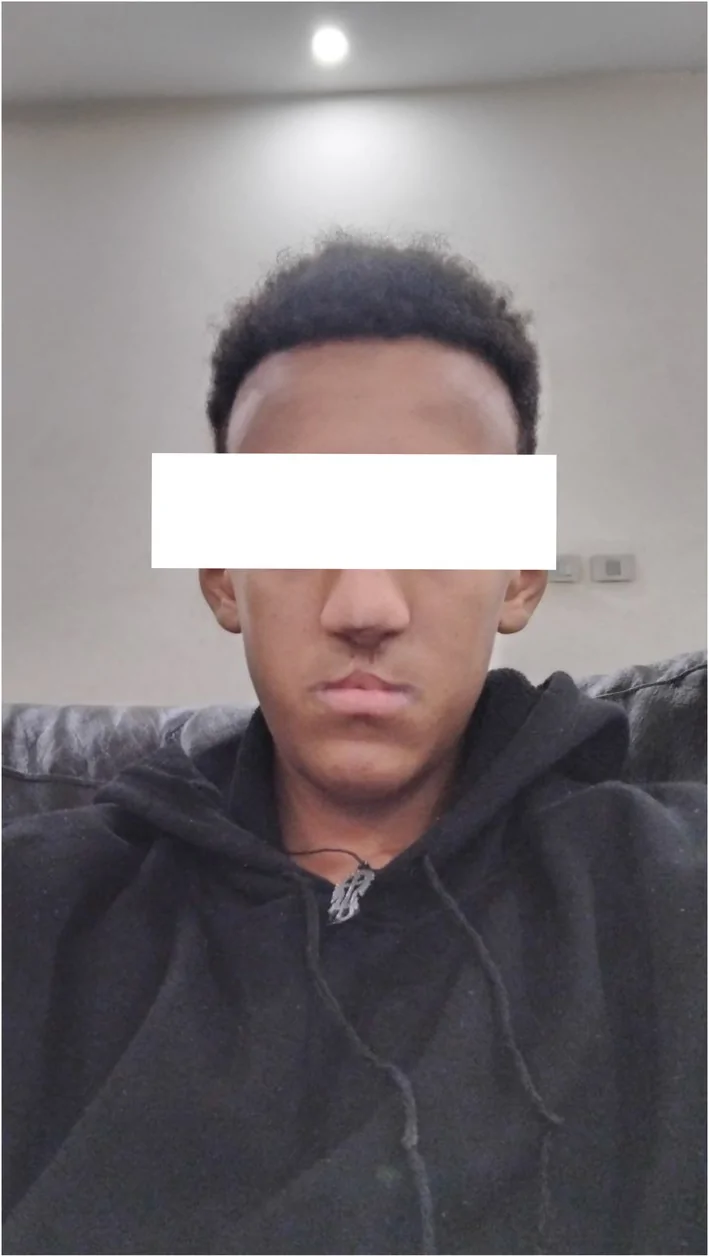
Postoperative photograph demonstrating mild residual facial and nasal soft tissue swelling following Caldwell–Luc surgery.

## 4 Discussion

Ectopic teeth are rare developmental anomalies characterized by the eruption or presence of a tooth in an abnormal anatomical location. Due to their uncommon nature, the true prevalence of ectopic teeth remains poorly defined, with available evidence primarily derived from isolated case reports and small case series. Several mechanisms have been proposed to explain their development, including disturbances in tooth germ formation, abnormal migration or displacement of dental tissues, genetic influences, and craniofacial developmental abnormalities such as cleft lip and palate. Other proposed contributing factors include dental trauma, maxillary osteomyelitis, congenital defects such as cleft palate, chronic nasal infections, and hereditary or genetic abnormalities that may interfere with normal tooth development and eruption pathways [1, 2, 3].

In our case, the most likely proposed etiology is the presence of cleft lip and palate. In patients with cleft lip and palate, abnormal development and fusion of the maxillary and alveolar processes can disrupt normal tooth development and eruption pathways, increasing the risk of ectopic tooth formation. These developmental disturbances may contribute to the occurrence of intranasal teeth, explaining their low but recognized prevalence among individuals with craniofacial anomalies [1, 4, 5].

Cleft lip and palate (CLP) is one of the most common orofacial congenital malformations, occurring in approximately 1 in 700 live births [6]. Patients with CLP often experience functional and esthetic challenges, and management may be complicated by associated dental abnormalities. Disturbances in maxillofacial development and tooth formation in CLP can result in various dental anomalies, including altered tooth number, morphology, position, and eruption patterns [7].

Several genetic and environmental factors, including gestational exposure to alcohol, cigarette smoking, steroids, rubella, anticonvulsants (phenobarbital and phenytoin), retinoids, and nutritional deficiencies, have been implicated in the development of CLP [8].

Dental anomalies in patients with CLP are more commonly observed on the cleft side and may involve both primary and permanent dentitions [9]. Among these abnormalities, ectopic tooth eruption has been reported in approximately 10.4% of patients with CLP, reflecting the impact of altered craniofacial development on normal eruption pathways [10]. However, ectopic teeth occurring in unusual locations, such as the nasal cavity, remain rare and are primarily described in isolated case reports. The development of ectopic teeth has been associated with abnormal tooth germ positioning, displacement of dental tissues, and craniofacial developmental disturbances.

The clinical presentation of intranasal ectopic teeth is variable. Many patients remain asymptomatic, and the anomaly may be discovered incidentally during imaging or examination. However, symptomatic patients may present with nasal obstruction, facial pain, headache, epistaxis, foul‐smelling rhinorrhea, external nasal deformity, or nasolacrimal duct obstruction. The differential diagnosis of a radiopaque intranasal lesion includes foreign bodies, rhinoliths, bone sequestration, neoplasms, and exostoses [11].

Diagnosis of intranasal ectopic teeth relies on a combination of clinical evaluation and radiologic imaging. Clinically, an intranasal tooth may appear as a hard white mass within the nasal cavity and can be surrounded by granulation tissue and debris due to chronic mucosal irritation. CT is considered the preferred imaging modality for evaluating sinonasal pathology, particularly for accurate localization and preoperative planning [12]. On CT, intranasal ectopic teeth appear as well‐defined radiopaque structures with attenuation similar to normal teeth. Bone window images may demonstrate a central radiolucent area corresponding to the pulp cavity, appearing as a spot or slit depending on the orientation of the tooth [13]. The characteristic dental morphology, including a recognizable crown, root, and pulp cavity, helps differentiate ectopic teeth from other radiopaque intranasal lesions. Unlike foreign bodies or rhinoliths, which typically appear as irregular calcified masses with heterogeneous mineralization and lack normal tooth architecture, ectopic teeth demonstrate an organized dental structure. Bone sequestra usually appear as irregular fragments of devitalized bone without a defined tooth pattern, while neoplasms may show calcification associated with soft tissue masses, bone destruction, or invasive features. Exostoses, in contrast, appear as smooth bony projections that maintain continuity with the adjacent cortex, whereas an ectopic tooth represents a separate dental structure. The surrounding soft tissue changes seen on CT may represent granulation tissue secondary to chronic irritation, correlating with clinical and histopathological findings [14, 15, 16].

Surgery is the main treatment option aimed to alleviate symptoms and prevent complications, such as osteomyelitis, nasal septal abscess or perforation, rhinosinusitis, dacryocystitis, oronasal or intraoral fistula, aspergillosis, and nasal deformity [17].

Several surgical approaches have been described for the removal of ectopic sinonasal teeth, including functional endoscopic sinus surgery (FESS), Caldwell–Luc antrostomy, and external approaches. FESS has become the preferred approach in many reported cases because it allows direct visualization through the natural nasal corridors, avoids external scars, preserves sinus drainage, and is associated with lower morbidity. However, FESS may be limited when the ectopic tooth is located anteriorly, embedded within the maxillary alveolar bone, or associated with altered anatomy. In our patient, previous cleft lip and palate repair had resulted in altered maxillofacial anatomy, including a residual left‐sided maxillary alveolar process and hard palate defect, which further limited straightforward endoscopic access. Therefore, a Caldwell–Luc approach was selected to provide direct exposure of the anterior maxillary region and facilitate controlled removal of the ectopic tooth while minimizing the risk of injury to adjacent structures [17, 18].

### 4.1 Strengths and Limitations

This case report highlights a rare occurrence of an intranasal ectopic tooth in an adolescent with repaired cleft lip and palate, emphasizing the diagnostic value of CT imaging. Detailed radiologic evaluation allowed precise localization of the ectopic tooth and facilitated successful surgical planning and management. The report contributes to the limited literature regarding the association between craniofacial anomalies and ectopic teeth.

However, as a single‐case report, the findings cannot be generalized to all patients with cleft lip and palate. Additionally, limited documentation regarding the patient's previous cleft repair, including the exact surgical technique, perioperative details, and early dental development, restricted a more comprehensive assessment of possible contributing factors.

## 5 Conclusion

Intranasal ectopic teeth are rare and require a high index of suspicion, as patients may be asymptomatic or present with nonspecific nasal symptoms. Because physical examination may not reliably identify the tooth due to overlying mucosa or anatomical distortion, CT plays a critical role in confirming the diagnosis, defining the exact location, detecting associated craniofacial abnormalities, and guiding surgical planning. Surgical removal remains the primary treatment to relieve symptoms and prevent potential complications.

## Author Contributions

**Mikyas Befekadu Nigusse:** conceptualization, investigation, writing – original draft, writing – review and editing, visualization, validation, methodology, formal analysis, supervision, data curation, project administration. **Bewuketu Kefyalew Zewudie:** conceptualization, investigation, methodology, resources, data curation, visualization, software, writing – original draft, writing – review and editing, formal analysis. **Ermias Algawork Kibru:** writing – review and editing, formal analysis, data curation, resources. **Nuhamin Daniel Kiflemariam:** investigation, methodology, formal analysis, data curation. **Bemnet Abi Zeleke:** investigation, validation, formal analysis, data curation.

## Funding

The authors have nothing to report.

## Ethics Statement

IRB review and approval were waived for this case report.

## Consent

Written informed consent was obtained from the patient for the publication of this case report and any accompanying images. A copy of the written consent is available for review by the Editor‐in‐Chief of this journal upon request.

## Conflicts of Interest

The authors declare no conflicts of interest.

## Data Availability

The data that support the findings of this study are available on request from the corresponding author. The data are not publicly available due to privacy or ethical restrictions.
